# Fornix white matter glia damage causes hippocampal gray matter damage during age-dependent limbic decline

**DOI:** 10.1038/s41598-018-37658-5

**Published:** 2019-01-31

**Authors:** Claudia Metzler-Baddeley, Jilu P. Mole, Rebecca Sims, Fabrizio Fasano, John Evans, Derek K. Jones, John P. Aggleton, Roland J. Baddeley

**Affiliations:** 1 Cardiff University Brain Research Imaging Centre (CUBRIC), Maindy Road, Cathays, Cardiff CF24 4HQ UK; 2 Psychological Medicine and Clinical Neurosciences, School of Medicine, Cardiff University, Maindy Road, Cathays, Cardiff CF24 4HQ UK; 3 Siemens Healthcare, Head Office, Sir William Siemens Square, Surrey, GU16 8QD UK; 4 School of Psychology, Faculty of Health Sciences, Australian Catholic University, Melbourne, Victoria 3065 Australia; 5 School of Psychology, Cardiff University, Tower Building, 70 Park Place, Cardiff, CF10 3AT UK; 6 Experimental Psychology, University of Bristol, 12a Priory Road, Bristol, BS8 1TU UK

**Keywords:** Cognitive ageing, Cognitive neuroscience, Glial biology

## Abstract

Aging leads to gray and white matter decline but their causation remains unclear. We explored two classes of models of age and dementia risk related brain changes. The first class of models emphasises the importance of gray matter: age and risk-related processes cause neurodegeneration and this causes damage in associated white matter tracts. The second class of models reverses the direction of causation: aging and risk factors cause white matter damage and this leads to gray matter damage. We compared these models with linear mediation analysis and quantitative MRI indices (from diffusion, quantitative magnetization transfer and relaxometry imaging) of tissue properties in two limbic structures implicated in age-related memory decline: the hippocampus and the fornix in 166 asymptomatic individuals (aged 38–71 years). Aging was associated with apparent glia but not neurite density damage in the fornix and the hippocampus. Mediation analysis supported white matter damage causing gray matter decline; controlling for fornix glia damage, the correlations between age and hippocampal damage disappear, but not *vice versa*. Fornix and hippocampal differences were both associated with reductions in episodic memory performance. These results suggest that fornix white matter glia damage may cause hippocampal gray matter damage during age-dependent limbic decline.

## Introduction

The world’s population is growing older and increasing numbers of people over the age of 65 will develop cognitive impairment due to late onset Alzheimer’s disease (LOAD)^1^. The pathological processes leading to LOAD accumulate over many years^2^ but prior to disease onset it remains challenging to reliably distinguish them from normal aging processes. Thus, recent recommendations of the Lancet commission^3^ highlight the importance of midlife prevention studies to gain a better understanding of the impact of aging and dementia risk in asymptomatic individuals.

Aging causes damage to both gray and white matter in the human brain. White matter consists of myelinated and unmyelinated axons and neuroglia cells, i.e., oligodendrocytes, astrocytes and microglia, while gray matter comprises neuronal cell bodies, synapses, dendrites and glia. Neuroglia cells are essential for normal synaptic and neuronal activity as they maintain the brain’s homeostasis, form myelin, protect neurons, and are dynamically remodelled to support brain plasticity^4–7^.

There are two classes of causal models of aging and LOAD risk: the first class of neurodegenerative models proposes that gray matter neuronal loss precedes white matter glia and axon damage. According to an influential neurodegenerative model, the amyloid cascade hypothesis^8,9^, aging and LOAD risk factors lead to metabolic changes in the amyloid precursor protein that result in the aggregation of amyloid-β plaques, which trigger pathological events including the formation of neurofibrillary tangles, loss of synapses, neurons and their axons. Immunity-related microglia changes occur in response to increased plaque and tangle burden^10–12^. The second class of model reverses the direction of causality, stating that the normal aging process in interaction with genetic and lifestyle risk factors of LOAD will cause neuroglia damage, resulting in impaired myelination, reduced microglia-mediated clearance and neuroinflammation, which in turn instigates pathological processes that lead to abnormal metabolism in key proteins and neuronal death^13–16^. The two classes of models predict opposite patterns of age and risk-related changes in white and gray matter. While in neurodegenerative models, white matter damage follows gray matter neuronal loss, the neuroglia model predicts that white matter neuroglia damage causes gray matter tissue loss.

**Figure 1:**
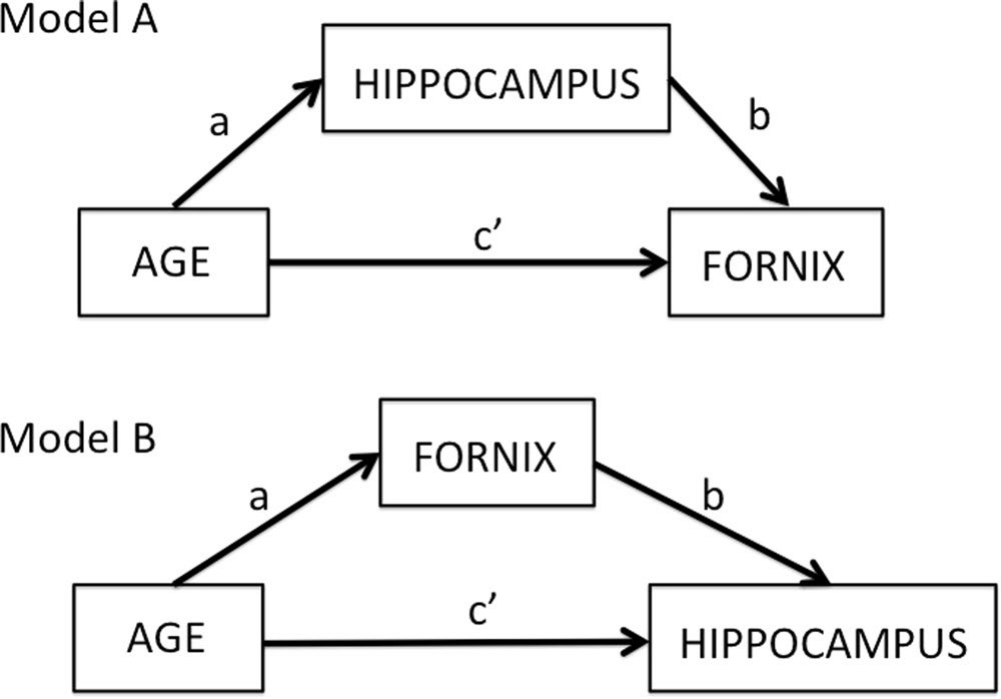
Overview of two mediation models: Model (**A**) tests for the indirect effects of hippocampal mediator variables on the direct effect of age on the fornix (path c’). The indirect effect is the product a*b of the correlations between age and hippocampus (path a) and the partial correlation between hippocampus and fornix accounting for age (path b). Model (**B**) tests the indirect effects of the fornix mediator variables on the direct effect of age on the hippocampus.

Currently, the nature of age-related white and gray matter tissue changes and the causal direction between them remain unknown. This study therefore investigated the impact of age and risk on quantitative MRI indices of apparent neurite and glia properties and discriminated between the two types of models using linear mediation analysis^17–19^. More specifically, we investigated the effects of aging and risk factors on tissue properties of two important gray and white matter structures involved in age-related memory decline, the hippocampus^20^ and the fornix^21^, in 166 asymptomatic individuals (39–71 years old) (Table 1). We then tested two mediation models displayed in Fig. 1: Model A assessed whether the inclusion of hippocampal variables mediated the direct effect of age on the fornix, whilst Model B tested whether fornix variables mediated age effects on the hippocampus. If full mediation of age effects was observed in Model A but not in Model B, then this would suggest that hippocampal gray matter differences were causing white matter fornix differences, consistent with neurodegenerative models of aging. However, if full mediation occurred in Model B but not Model A, then this would suggest that age-related fornix differences were causing hippocampal gray matter differences, consistent with neuroglia models of aging.

**Table 1:** Summary of demographic, genetic and lifestyle risk information of participants.

Mean (SD)	Participants (n = 166)
Age (in years)	55.8 (8.2)
Females	56%
Years of education	16.5 (3.3)
NART	116.7 (6.7)
MMSE	29.1 (1.0)
Rey figure immediate recall	25.7 (5.5)
Rey figure delayed recall	24.2 (5.8)
RAVLT immediate recall	8.8 (1.9)
RAVLT delayed recall	12.1 (2.3)
Positive Family History dementia	35.1%
*APOE* Genotype %	
ε2/ε2	0.6%
ε2/ε3	10.7%
ε2/ε4	3.6%
ε3/ε3	48.8%
ε3/ε4	32.1%
ε4/ε4	4.2%
Abdominal obesity (Waist Hip Ratio)	61.3%
Systolic Hypertension	28%
Smokers	5.4%
Diabetes	1.8%
Statins	7.2%
Alcohol units per week	7.4 (9.3)
Physical activities^*^	10.3 (12.1)
(Median hours per week)	

^*^Based on all non-sedentary activities including walking, house work, gardening.

*APOE* = Apolipoprotein-E, MMSE = Mini Mental State Exam, NART = National Adult Reading Test, RAVLT = Rey Auditory Verbal Learning Test.

**Figure 2:**
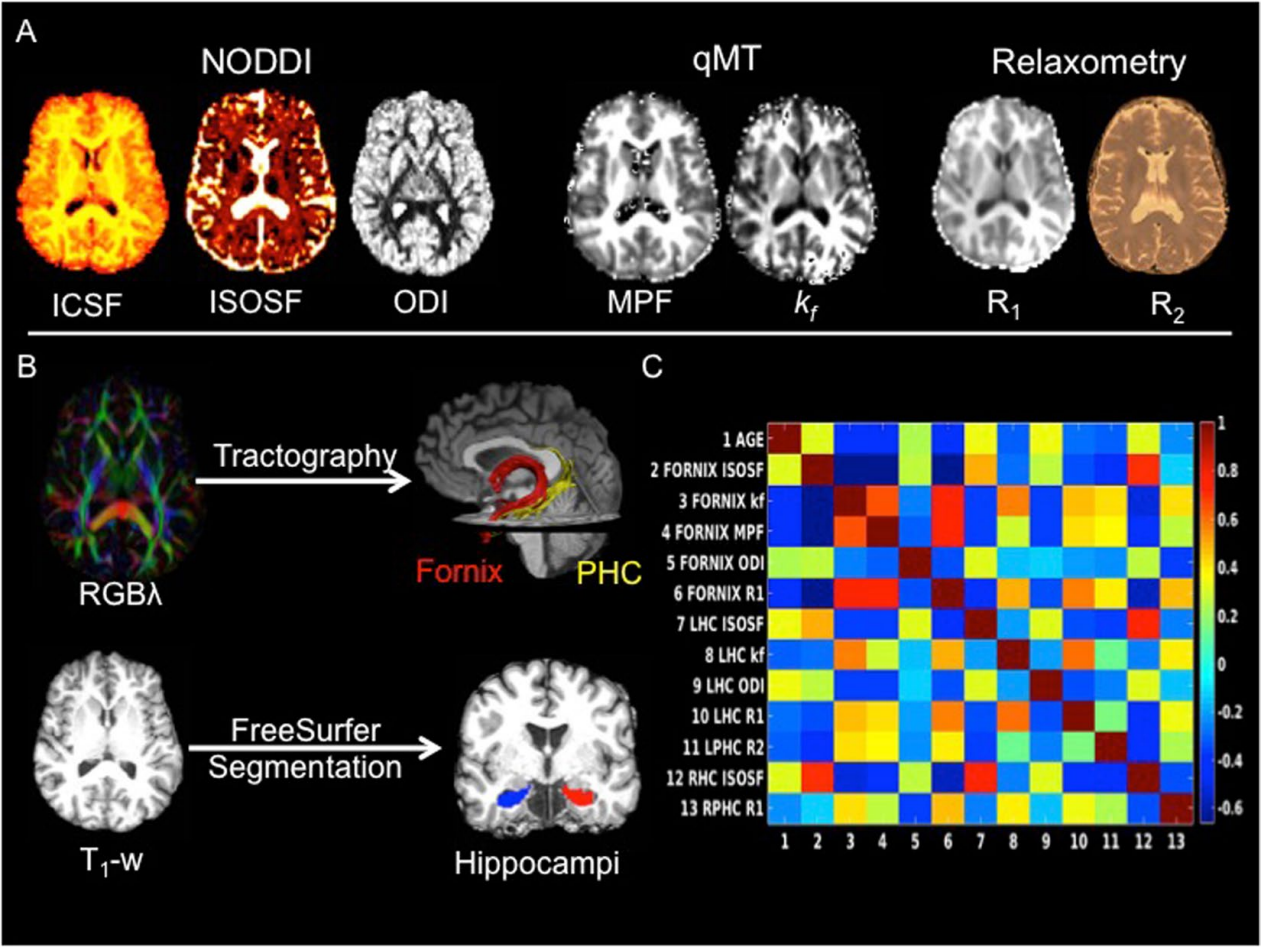
(**A**) MRI modalities and maps acquired from dual-shell high angular resolution diffusion imaging (HARDI), quantitative magnetisation transfer (qMT) imaging and from T_1_ and T_2_ relaxometry. HARDI data were modelled with neurite orientation dispersion and density imaging (NODDI), yielding maps of intracellular signal fraction (ICSF), isotropic signal fraction (ISOSF) and orientation dispersion index (ODI). The qMT maps were the macromolecular proton fraction (MPF) and the forward exchange rate *k*_f_. Maps from relaxometry were the longitudinal relaxation rate R_1_ (1/T_1_) and R_2_ (1/T_2_). (**B**) Mean indices of the metrics were extracted from left and right hippocampi, fornix and parahippocampal cinguli (PHC) tracts. Hippocampi were segmented from T_1_- weighted images with FreeSurfer version 5.3 and fornix and PHC were reconstructed with damped-Richardson Lucy spherical deconvolution (dRL) based deterministic tractography on colour coded principal direction maps (RGBλ). (**C**) Cross-correlation matrix between white and gray matter microstructural indices that correlated with age.

We employed multi-modal quantitative MRI measurements to assess gray and white matter tissue properties in the hippocampus, the fornix, and the parahippocampal cingulum (PHC), as a temporal lobe comparison pathway. Separate estimates of neurite and glia-related microstructural tissue properties were obtained from diffusion neurite orientation dispersion and density imaging (NODDI)^22^, quantitative magnetization transfer (qMT)^23–30^ and relaxometry imaging (Fig. 2A).

NODDI yielded the intracellular signal fraction (ICSF), an index of apparent neurite density, the neurite orientation dispersion index (ODI), and the isotropic signal fraction (ISOSF), an estimate of free water contribution to the diffusion signal. Diffusion MRI metrics are well-known measures of white matter microstructure^31–33^ and NODDI ICSF and ODI have been proposed to be particularly sensitive to axon density and dispersion^34,35^. In addition, we used qMT, a technique that allows the quantification of differences in brain macromolecular density, and provides indices with improved white matter glia specificity compared to diffusion MRI^27–30^. In white matter, magnetization transfer is dominated by myelin^36,37^, and is sensitive to microglia-mediated inflammation^28,38,39^. We measured the macromolecular proton fraction (MPF) as an index of white matter neuroglia that will largely reflect its myelin content and the forward exchange rate *k*_*f*_, an index of the rate of the magnetization transfer process^27^, that has been proposed to reflect metabolic efficiency of mitochondrial function^40^ (Fig. 2A). Finally, the longitudinal relaxation rates R_1_ (1/T_1_) and R_2_ (1/T_2_) were obtained as additional estimates of the relative contribution of water, myelin and iron content of gray and white matter tissue^41–44^ (Fig. 2A).

Mean values of all MRI indices were extracted from the fornix and the PHC tracts, that were reconstructed with deterministic tractography, and from FreeSurfer segmentations of the whole hippocampi^45,46^ (Fig. 2B). The hippocampal regions included areas of the presubiculum, subiculum, cornu ammonis subfields 1–4, dentate gyrus, hippocampal tail and fissure but excluded cortical regions such as the entorhinal cortex^47,48^.

To study the relationships between age-related differences in MRI metrics and genetic and lifestyle risk factors of LOAD as well as differences in episodic memory performance, we also acquired the following information (Table 1): Genetic risk was assessed by carriage of the Apolipoprotein-E (*APOE*) ε4 allele^49,50^ and separately by a positive family history of a first grade relative with dementia of the Alzheimer’s, Lewy body or vascular type. We predicted more pronounced white matter glia reductions in *APOE* ε4 compared to ε2 and/or ε3 carriers, as cholesterol transport for myelin repair in the brain has been proposed to be less efficient in ε4 carriers^50–53^. In addition, we assessed lifestyle-related risk factors associated with the metabolic syndrome, notably central obesity, hypertension, alcohol consumption and sedentary lifestyle^54,55^ (Table 1). We were particularly interested in the effects of central adiposity, as obesity is globally on the rise, and is associated with chronic inflammation, insulin resistance and vascular problems^54,55^ as well as with accelerated aging in brain regions that include limbic white matter^56,57^. Finally, episodic memory performance was assessed with standard neuropsychological tests of verbal and non-verbal recall^58,59^ (Table 1).

Neuroglia models predict that aging and risk factors will primarily reduce glia sensitive metrics of MPF, *k*_*f*_, R_1_ and R_2_ whilst apparent neurite density (ICSF) and dispersion (ODI) should be less affected. In contrast, neurodegenerative models predict impairments in ICSF and ODI with relatively preserved glia-metrics. Age and risk-related increases in ISOSF, reflective of increased free water content in brain tissue, may be expected by both models. Importantly though, neurodegenerative models predict that hippocampal differences will mediate age-related white matter microstructural differences in the fornix (Model A), whilst neuroglia models predict that age-related differences in glia sensitive white matter metrics of the fornix will mediate age-related hippocampal differences (Model B).

## Results

### Omnibus multivariate regression analysis

Multivariate regression analysis with all hippocampal, fornix and PHC MRI outcome metrics as dependent variables, tested simultaneously for omnibus effects of the following independent variables:

- age,
- genetic risk: family history of dementia (yes/no), *APOE* genotype [ε2 (ε2/ε2, ε2/ε3), ε3(ε3/ε3), ε4(ε3/ε4, ε4/ε4)],
- lifestyle-related risk: central obesity assessed with the waist hip ratio (WHR), systolic and diastolic blood pressure (BP), weekly alcohol consumption and physical activity,
- and potentially confounding variables of sex, years of education, and head size assessed with intracranial volume (ICV).

Given the low numbers of diabetics, smokers, and individuals on statins amongst our sample (Table 1), these variables were not included in the analyses. This analysis revealed significant omnibus effects of age [F(35, 96) = 3.0, p = 0.000015; ηp^2^ = 0.52] and ICV [F(35, 96) = 2.3, p = 0.001, ηp^2^ = 0.45].

**Figure 3:**
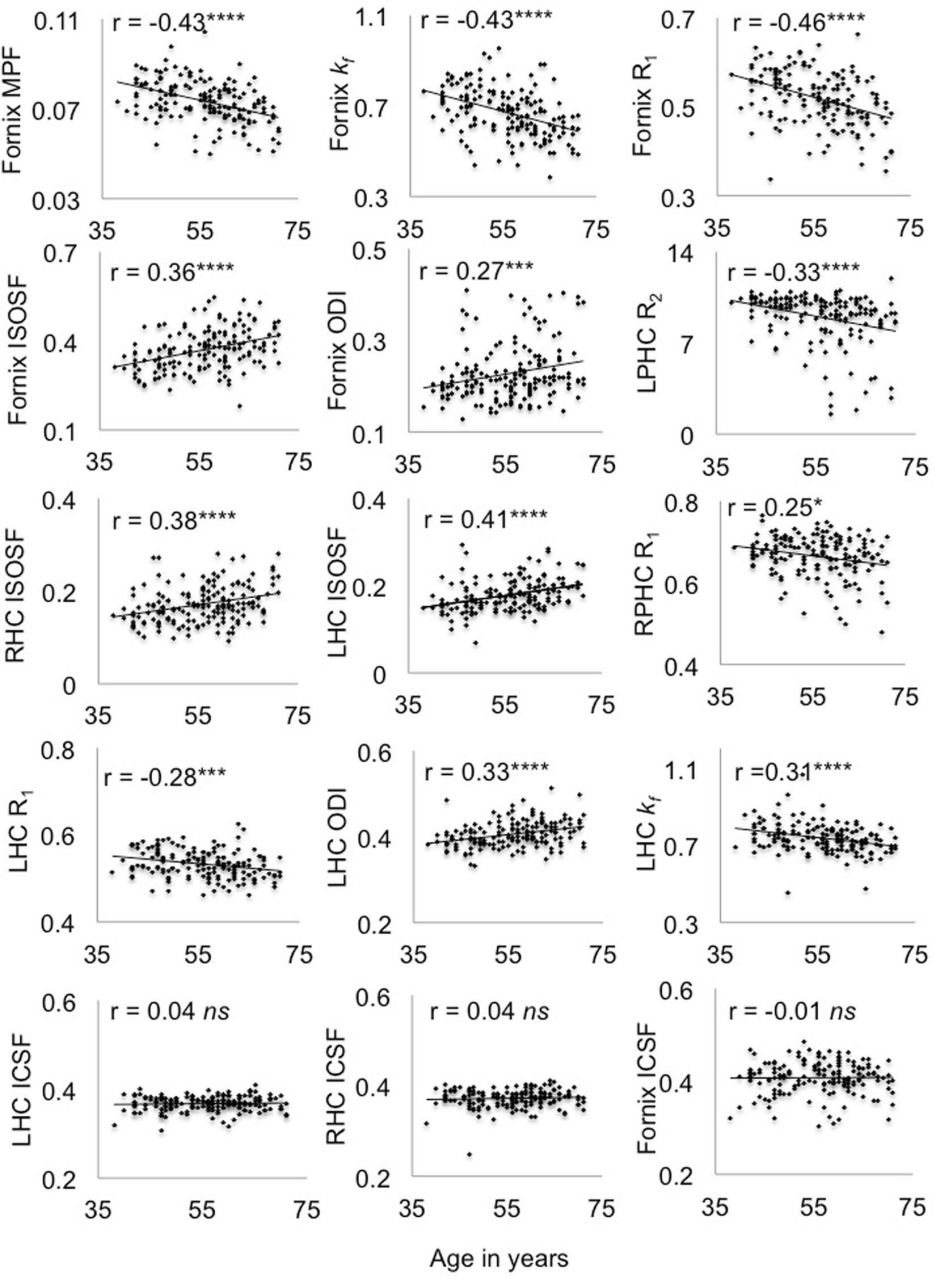
Plots the correlations and Pearson coefficients (controlled for intracranial volume) between age and white and gray matter microstructural indices. Abbr.: ICSF = intracellular signal fraction, ISOSF = isotropic signal fraction, *k*_*f*_* = *forward exchange rate, LHC = left hippocampus, LPHC = left parahippocampal cingulum, MPF = macromolecular proton fraction, ODI = orientation dispersion index, R = longitudinal relaxation rate, RHC = right hippocampus, RPHC = right parahippocampal cingulum ****p < 0.0001, ***p < 0.001, **p < 0.01, *p < 0.05, 5% False Discovery Rate corrected.

*Post-hoc* multivariate covariance analyses, controlling for ICV, then tested for the effects of age on each of the hippocampal, fornix and PHC MRI indices separately. Table 2 summarises the age effects on white and gray matter microstructure. Age affected all fornix indices, except ICSF [F(2, 152) = 1.1, p = 0.35]. There were also significant age effects for left PHC R_2_, right PHC R_1_ and for left and right hippocampal ISOSF, left hippocampal R_1_, *k*_*f*_ and ODI (Table 2) (Fig. 3). No age effects were observed for hippocampal ICSF [left: F(2, 152) = 0.2, p = 0.84; right: F(2, 152) = 0.7, p = 0.48] (Fig. 3).

**Table 2:** Summary of the effects of age on gray and white matter microstructural indices.

MRI index		F_(2,152)_-value^*^	p-value^**^	Effect size ηp^2^
Fornix	MPF	11.9	0.00002	0.14
	*k*_*f*_	10.0	0.00009	0.12
	R_1_	12.4	0.00001	0.14
	ISOSF	8.9	0.0002	0.11
	ODI	5.0	0.008	0.06
Left PHC	R_2_	7.5	0.001	0.09
Right PHC	R_1_	4.7	0.01	0.06
Left hippocampus	*k*_*f*_	6.7	0.002	0.08
	R_1_	5.0	0.008	0.06
	ISOSF	12.2	0.00001	0.14
	ODI	9.8	0.0001	0.12
Right hippocampus	ISOSF	7.5	0.001	0.09

^*^Controlled for intracranial volume, **5% FDR corrected. ISOSF = isotropic signal fraction, MPF = macromolecular proton fraction, ODI = orientation dispersion index, PHC = parahippocampal cingulum.

Age correlated negatively with fornix MPF, R_1_, *k*_*f*_, left PHC R_2_, right PHC R_1_, left hippocampal *k*_*f*_ and R_1_, and positively with fornix and hippocampal ISOSF and ODI (Fig. 3). No age correlations were present with ICSF in the fornix or the hippocampi and these ‘null’ correlations differed significantly from the smallest age correlations in the fornix (z_ODI_ = 2.5, p = 0.01), left hippocampus (z_R1_ = 2.78, p = 0.005) and right hippocampus (z_ISOSF_ = 3.18, p = 0.002) (Fig. 3).

Figure 2C displays the Pearson correlation coefficient matrix between the white and hippocampal gray matter metrics. The same metrics measured in white and gray matter correlated positively with each other with the exception of fornix and left hippocampal ODI (r = −0.12, p = 0.13). MPF, *k*_*f*_, R_1_ and R_2_ correlated positively with each other and so did ISOSF and ODI, whilst MPF, *k*_*f*_, R_1_ and R_2_ were inversely correlated with ISOSF and ODI.

### Mediation analyses between hippocampal and fornix metrics

To avoid any bias in the comparison of Model A and Model B, the number of mediator variables, that showed significant age effects and correlated with each other in white and gray matter, was kept constant. Mediator variables in Model A were the left and right hippocampal ISOSF, left hippocampal R_1_ and left hippocampal *k*_*f*_, and in Model B the four white matter metrics that showed the largest age effects, i.e., fornix ISOSF, MPF, *k*_*f*_, and R_1_. There were no significant differences between the age effect sizes in the fornix and the hippocampus (r_max:age-fornix R1_ *versus* r_min:age-left hippocampal R1_: z = 1.8, p = 0.07).

**Figure 4:**
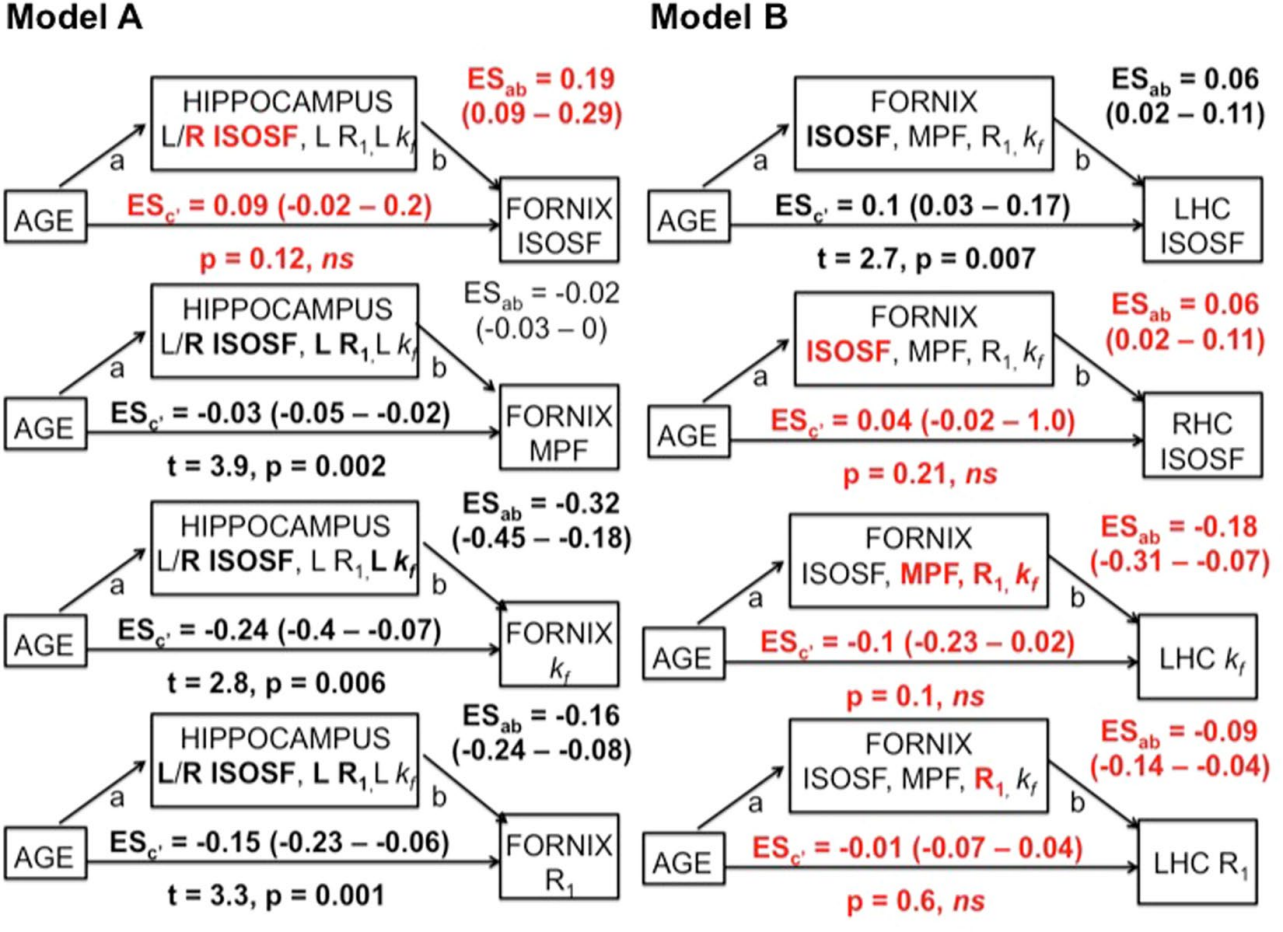
Summarises the results of the mediation analyses for Models **A** and **B**. 95% confidence intervals of the effect sizes (ES × 10^−1^) were based on bootstrapping with 5000 replacements. Fornix mediators in Model B had significant indirect effects and fully mediated the direct age effects on right hippocampal (RHC) isotropic signal fraction (ISOSF), left hippocampal (LHC) R_1_ and *k*_*f*_ (highlighted in red). In contrast, hippocampal mediators, although showing significant indirect effects (highlighted in bold), did not fully mediate the direct age effects on fornix MPF, R_1_ and *k*_*f*_. Right (R) hippocampal ISOSF fully mediated fornix ISOSF and *vice versa* but fornix mediators did not remove the age effect on left hippocampal ISOSF. Mediator variables that contributed significantly to the regression analyses after 5% FDR correction are highlighted in bold.

In Model B, fornix mediator variables fully mediated the age effects on right hippocampal ISOSF, left hippocampal *k*_*f*_ and left hippocampal R_1_ (Fig. 4, highlighted in red) but hippocampal mediators in model A, although showing significant indirect effects on the direct effects of age on fornix R_1_ and *k*_*f*_, did not remove the age effects on fornix MPF, R_1_ and *k*_*f*_. A bi-directional relationship was observed between right hippocampal ISOSF and fornix ISOSF, with both variables fully mediating each other’s age effect. Finally, fornix ISOSF contributed but did not fully mediate the correlation between age and left hippocampal ISOSF, and left hippocampal ISOSF only marginally contributed to the age correlation in fornix R_1_. To summarise, full mediation of age effects on glia metrics was observed in Model B but not in Model A (Fig. 1), such that age-related glia differences in the fornix mediated age differences in the hippocampus but not *vice versa*.

### Effects of genetic and lifestyle risk factors on age-mediator variables

We further explored whether genetic and lifestyle risk factors had an effect on mediator variables that fully accounted for age effects with hierarchical regression analysis. First age, ICV, sex and years of education were entered into the regression model followed by the stepwise inclusion of all genetic and lifestyle risk variables. Table 3 summarises the results of these regression analyses. Besides age, sex was a significant predictor for differences in fornix and hippocampal ISOSF. WHR contributed significantly to fornix MPF and R_1_ and alcohol consumption to fornix R_1_. There were trends (significant at the uncorrected level) for a contribution of family history of dementia and diastolic BP to differences in right hippocampal ISOSF.

**Table 3:** Summary of the results of the hierarchical regression models testing for the effects of genetic and lifestyle risk variables on fornix and hippocampus mediator variables.

Outcome variables	Adjusted R^2^	Predictors in final regression model
Fornix MPF	**0.24 (p** < **0.001)**	**Age (p** < **0.001)**
		**WHR (p** = **0.02)**
Fornix R_1_	**0.28 (p** < **0.001)**	**Age (p** < **0.001**)
		**ICV (p** = **0.009)**
		**Alcohol (p** = **0.01)**
		**WHR (p** = **0.018)**
Fornix k_f_	**0.23 (p** < **0.001)**	**Age (p** < **0.001)**
		WHR (p = 0.04)
Fornix ISOSF	**0.32 (p** < **0.001)**	**Age (p** < **0.001)**
		ICV (p = 0.03)
		**Sex (p** = **0.001)**
Right hippocampal ISOSF	**0.36 (p** < **0.001)**	**Age (p** < **0.001)**
		**ICV (p** = **0.008)**
		**Sex (p** = **0.002)**
		Diastolic BP (p = 0.035)
		FH (p = 0.04)

5% FDR corrected p-values are highlighted in bolds.

*Post-hoc* comparisons revealed that centrally obese individuals compared with individuals with a normal WHR showed lower MPF [t(160) = 2.8, p = 0.005] and R_1_ [t(160) = 3.35, p = 0.0009] in the fornix. Men compared with women showed higher ISOSF in the right hippocampus [t(162) = 6.5, p < 0.00001] and in the fornix [t(164) = 6.7, p < 0.00001]. Participants with a positive family history exhibited higher ISOSF in the right hippocampus [t(160) = 2.55, p = 0.012] and there was a positive correlation between diastolic BP and ISOSF in the right hippocampus [r(164) = 0.18, p = 0.02]. However, there was no difference in fornix R_1_ between individuals that consumed alcohol units above the weekly-recommended limit compared with those within the UK recommended guidelines (p = 0.9).

### Correlations between age-mediator variables and episodic memory performance

Inter-individual differences in delayed verbal recall were negatively correlated with differences in fornix ISOSF [r(164) = −0.22, p = 0.005) and differences in right hippocampal ISOSF [r(164) = − 0.2, p = 0.006].

### CSF partial volume effects

To test whether age effects on fornix and hippocampal qMT metrics were driven by free water partial volume effects, ISOSF metrics were used as mediator variables. Fornix ISOSF contributed significantly to all regression models (p < 0.0001) but did not remove the direct age effect on fornix MPF (t = −3.88, p = 0.0002), R_1_ (t = −3.69, p = 0.0003), and *k*_*f*_ (t = −3.88, p = 0.0002). Right but not left hippocampal ISOSF contributed significantly to left hippocampal R_1_ (p = 0.003) and *k*_*f*_ (p = 0.03) and removed the direct age effect on left hippocampal R_1_ (p = 0.05) but not on left hippocampal *k*_*f*_ (p = 0.0013).

## Discussion

The aim of this study was to discriminate between two classes of causal models of aging with mediation analysis. The neurodegenerative model predicted that age-related hippocampal differences would account for age effects on fornix metrics whilst the neuroglia model predicted that age differences in fornix glia would cause age-differences in the hippocampus. Our results are consistent with the neuroglia model as we found that fornix glia sensitive metrics of MPF, R_1_ and *k*_*f*_ fully mediated the effects of age on hippocampal tissue properties but not *vice versa*, i.e., hippocampal mediator variables, although demonstrating significant indirect effects, did not remove the age effects on fornix MPF, R_1_ and *k*_*f*_ (Fig. 4). This pattern of results is consistent with a growing body of evidence pointing towards an important role of neuroglia changes in aging and neurodegeneration^11,13–15,60–62^. However, to the best of our knowledge, this is the first study to show that fornix white matter glia damage may cause hippocampal gray matter damage during age-dependent limbic decline in the human brain.

Both in the hippocampus and the fornix, we observed age-related increases in ISOSF and ODI and reductions in glia-sensitive metrics from qMT and relaxometry imaging, whilst age had no effects on ICSF. Overall this pattern of results suggests age-dependent i) increases in the free water content of tissue, ii) reductions in glia-sensitive indices, and iii) increases in apparent neurite dispersion in the absence of differences in apparent neurite density.

In white matter, MPF, *k*_*f*_, R_1_ and R_2_ are known to be sensitive to myelin, as well as to neuroinflammation and to a lesser degree to iron changes^27–30,38,44,63,64^. While some effects were observed on R_1_ and R_2_ in the PHC, the largest age-dependent reductions in MPF, *k*_*f*_, and R_1_ were present in the fornix tract. Reductions in these indices in the absence of any effects on ICSF suggest that aging affects glia-sensitive properties of white matter, notably myelin, but not apparent axon density, i.e. not the number and the size of axons. These results provide novel *in vivo* neuroimaging evidence consistent with neuropathological findings of a reduction of up to 45% of the length of myelinated fibers, rather than a loss of axons *per se* across the lifespan^65–69^. Similarly, age-related reductions in fornix myelination and glia changes in the absence of any changes of the fornix cross-sectional area were also observed in non-human primates^70^. Myelin damage is closely linked to neuroinflammation and qMT metrics are sensitive to both processes^71^. Thus it may also be possible that the observed differences in MPF and *k*_*f*_ reflect age-dependent dystrophic and reactive microglia changes^39,72^.

Similarly, in hippocampal gray matter we observed age-related reductions of R_1_ and *k*_*f*_ in the absence of differences in ICSF. Age reductions in hippocampal R_1_ were accounted for by increases in hippocampal ISOSF and may, therefore, primarily reflect increases in the water content of hippocampal tissue. Increases in hippocampal ISOSF are consistent with neuropathological findings of a loss of hippocampal pyramidal cells and dentate gyrus granule cells associated with normal aging^73^. However, the absence of an effect on hippocampal ICSF suggests that apparent dendritic density was unaffected and increased ISOSF may also be compatible with glia-related changes in gray matter. Furthermore, the observed reductions in hippocampal *k*_*f*_, a marker of the rate of the magnetization transfer between macromolecular and free water pool, may reflect age-dependent metabolic differences. Giulietti *et al*.^40^ found *k*_*f*_ reductions in the hippocampus, temporal lobe, posterior cingulate and parietal cortex in LOAD, and proposed that these changes may reflect reductions in metabolic mitochondrial activity^74^. Age-related reductions of hippocampal metabolic activity have also been observed in animal studies^75,76^ and age-related damage to mitochondria of microglia has been linked to sustained microglia neuroinflammation^7^.

Currently, a causal link between age-related neuroglia changes and the development of LOAD pathology in the human brain remains speculative. However, white matter disease, characterised by a loss of myelin, oligodendrocytes, axons and reactive astrocyte gliosis^77–79^ is a known feature of LOAD. Accumulating evidence also suggests that neuroinflammation and a reduction of glia mediated clearance mechanisms contribute to LOAD^11,12,80^. For instance, reactive microglia were found in the hippocampus of LOAD brains^81^ and microglia derived ASC protein specks have been shown to cross-seed amyloid-β plaques in transgenic double-mutant APPSwePSEN1dE9 mice^82^. It is therefore possible that age-related neuroglia changes involving myelin and inflammation pathways may not only occur in response to neuropathology but may even trigger protein abnormalities and synaptic and neuronal loss. Thus, an intriguing interpretation of our results may be that aging is associated with glia changes including loss of myelin in the fornix, which in turn may cause tissue damage in the hippocampus, that might make this region more vulnerable to the development of LOAD pathology^81,83^. Clearly, future longitudinal prospective studies assessing the predictive value of MRI indices of white matter glia for amyloid and tau burden are needed to test this hypothesis.

We also observed a bi-directional mediation effect between right hippocampal ISOSF and fornix ISOSF, such that both variables fully mediated the age effect on each other. This result is unsurprising and reflects that unspecific tissue loss in the hippocampus is associated with tissue loss in the fornix and the other way around. In contrast, fornix and left hippocampal ODI metrics were not correlated with each other. This finding suggests that ODI indices may capture different microstructural properties and may not be directly comparable in gray and in white matter, potentially due to differences in tissue complexity and organisation.

The question arises how genetic and lifestyle risk factors of LOAD impact on medial temporal tissue properties. Consistent with our hypothesis, centrally obese individuals exhibited reductions in fornix MPF and R_1_. Obesity-related reductions in MRI metrics of proton density, R_1_, and R_2_^*^, have previously been observed in white matter pathways connecting frontal and limbic regions in young adults^84^. Obesity has also been associated with accelerated aging of cerebral white matter^56^ and we previously reported Body Mass Index related increases in mean and axial diffusivities in the fornix^57^. Although the mechanisms underpinning these changes remain unclear, it may be possible that the observed MRI differences may reflect changes in neuroglia that may be linked with obesity-related systemic inflammation. Consistent with this interpretation, differences in white matter fractional anisotropy in obesity were reported to be associated with inflammation and to a lesser degree with glucose regulation, whilst lower blood pressure and dyslipidemia appeared to have positive effects on white matter microstructure^85^.

Unexpectedly and in contrast to previous reports^52,53,86^ we did not observe a main effect of the *APOE* genotype on hippocampal gray matter or fornix/PHC white matter tissue properties. There is substantial evidence that *APOE* ε4 carrier status in older individuals (>65 years of age) is related to accelerated atrophy in the hippocampus, increased beta-amyloid burden and to an earlier onset of dementia^87,88^. However, the effect size of *APOE-*ε4 genotype on age-related hippocampal atrophy over the adult life course is small (e.g. β = −0.05, p = 0.04 in n = 3749 between 43–69 years of age)^89,50,90–93^ and the biological mechanisms underpinning these relationships remain poorly understood. The *APOE* gene is involved in many complex functions, including lipid and amyloid-β metabolism, neuroinflammation and vascular regulation^94^. It is likely that *APOE* genotype, rather than exhibiting straightforward main effects, interacts with age and with multiple genetic and lifestyle-related factors. As the effects of *APOE* genotype were not the main focus of this study, testing for potential interaction effects between *APOE* genotype and other demographic and risk factors was beyond the scope of this paper.

In contrast to *APOE*, we observed some contribution of family history of dementia and diastolic blood pressure to hippocampal ISOSF, but these effects did not survive multiple comparison correction. We also found that men compared with women showed larger ISOSF in the right hippocampus and in the fornix. These results are compatible with previous findings, indicating that both family history^95^ and being male^96–99^ are associated with accelerated age-related atrophy of cortical and subcortical brain regions, including the hippocampus and the fornix.

Finally, we found evidence that the observed age-dependent decline in limbic structures had some impact on individual differences in episodic memory performance, as individual differences in fornix and hippocampal ISOSF were negatively correlated with differences in delayed verbal recall performance. This finding is consistent with a large body of evidence demonstrating an important role of the hippocampal-fornix axis in mediating age-dependent decline of episodic memory function^21^.

In conclusion, we provide novel evidence in support of the neuroglia model of aging. We propose that age-related damage to fornix glia, rather than a loss of axons, may cause hippocampal tissue changes associated with reduced metabolism, glia and neuronal loss. These results provide novel insights into the mechanisms underpinning age-dependent decline in limbic structures and suggest that MRI metrics sensitive to neuroglia may potentially provide useful biomarkers of midlife risk of LOAD.

## Methods

This study was approved by the Cardiff University Psychology Research Ethics Committee (EC.14.09.09.3843R2). All participants provided written informed consent in accordance with the Declaration of Helsinki.

### Participants

Community-dwelling participants between 35 and 75 years of age were recruited from Cardiff University panels and notice boards and *via* internet and poster advertisements. Exclusion criteria included a history of neurological or psychiatric disease, head injury with loss of consciousness, drug/alcohol dependency, high-risk cardio-embolic source, large-vessel disease, and MRI contraindications. From 211 recruited volunteers, 166 underwent MRI scanning at CUBRIC. Table 1 summarises the demographic, cognitive, and dementia risk information for these 166 participants.

### Assessment of genetic and lifestyle related risk factors

#### Genetic-related risk

Saliva samples were collected with the Oragene-DNA (OG-500) kit (Genotek). APOE genotypes ε2, ε3 and ε4 were determined by TaqMan genotyping of single nucleotide polymorphism (SNP) rs7412 and KASP genotyping of SNP rs429358. Genotyping was successful in 165 individuals that underwent an MRI scan (Table 1). Participants also provided information about their family history of dementia, i.e. whether a first-grade relative was affected by LOAD, vascular dementia or Lewy body disease with dementia.

#### Lifestyle-related risk

Abdominal adiposity was assessed with the waist-hip-ratio (WHR)^100^. Central obesity was defined as WHR ≥ 0.9 for men and ≥ 0.85 for women. Systolic and diastolic blood pressure was measured with a digital blood pressure monitor (Model UA-631; A&D Medical, Tokyo, Japan). Hypertension was defined as systolic BP ≥ 140 mm Hg. Risk factors of diabetes mellitus, high levels of blood cholesterol, history of smoking and weekly alcohol intake were self-reported. Information about participants’ physical activity over the previous week was collected with the International Physical Activity Questionnaire (IPAQ)^101^. Participants’ intellectual and cognitive functions were assessed with the National Adult Reading Test (NART)^102^ and the Mini Mental State Exam (MMSE)^103^, episodic memory abilities with the Rey Auditory Verbal Learning test (RAVLT)^58,59^ and the Rey Complex Figure^65^, and depression with the Patient Health Questionnaire for Depression (PHQ-9)^104^.

### MRI data acquisition

MRI data were acquired on a 3 T MAGNETOM Prisma clinical scanner (Siemens Healthcare, Erlangen, Germany) equipped with a 32-channels receive-only head coil at CUBRIC.

#### Anatomical MRI

T_1_-weighted anatomical images were acquired with a three-dimension (3D) magnetization-prepared rapid gradient-echo (MP-RAGE) sequence with a 256 × 256 acquisition matrix, TR = 2300 ms, TE = 3.06 ms, TI = 850 ms, flip angle θ = 9°, 176 slices, 1 mm slice thickness, FOV = 256 mm, and acquisition time of ~6 min.

#### High Angular Resolution Diffusion Imaging (HARDI)

Diffusion data (2 × 2 × 2 mm voxel) were collected with a spin-echo echo-planar dual shell HARDI^105^ sequence with diffusion encoded along 90 isotropically distributed orientations (30 directions at b-value = 1200 s/mm^2^ and 60 directions at b-value = 2400 s/mm^2^) and six non-diffusion weighted scans with dynamic field correction, TR = 9400 ms, TE = 67 ms, 80 slices, 2 mm slice thickness, FOV = 256 × 256 × 160 mm, GRAPPA acceleration factor = 2 and acquisition time of ~15 min.

#### Quantitative magnetization transfer weighted imaging (qMT)

An optimized 3D MT-weighted gradient recalled-echo sequence^106^ was used to obtain magnetization transfer-weighted data with TR = 32 ms, TE = 2.46 ms; Gaussian MT pulses, duration t = 12.8 ms; FA = 5°; FOV = 24 cm, and 2.5 × 2.5 × 2.5 mm^3^ resolution. The following off-resonance irradiation frequencies (Θ) and their corresponding saturation pulse nominal flip angle (ΔSAT) for the 11 MT-weighted images were optimized using Cramer-Rao lower bound optimization: Θ = [1000 Hz, 1000 Hz, 2750 Hz, 2768 Hz, 2790 Hz, 2890 Hz, 1000 Hz, 1000 Hz, 12060 Hz, 47180 Hz, 56360 Hz] and their corresponding ΔSAT = [332°, 333°, 628°, 628°, 628°, 628°, 628°, 628°, 628°, 628°, 332°]. The longitudinal relaxation time, T_1_, of the system was estimated by acquiring three 3D gradient recalled echo sequence volumes with three different flip angles (θ = 3°, 7°, 15°) using the same acquisition parameters as used in the MT-weighted sequences (TR = 32 ms, TE = 2.46 ms, FOV = 24 cm, 2.5 × 2.5 × 2.5 mm^3^ resolution). Static magnetic field maps (B_0_) were collected using two 3D GRE volumes with different echo-times (TE = 4.92 ms and 7.38 ms respectively; TR = 330 ms; FOV = 240 mm; slice thickness 2.5 mm)^107^.

#### T_2_-weighted maps

were acquired with a multi-echo spin echo sequence with five equally spaced echo times (TEs = 13.8 ms – 69 ms), TR = 1600 ms, 19 slices, 5 mm slice thickness, FOV = 240 mm, flip angle θ = 180° and acquisition time of ~6 min.

### MRI data processing

The HARDI data were corrected for distortions induced by the diffusion-weighted gradients and artifacts due to head motion with reorientation of the encoding vectors^108^ in ExploreDTI (Version 4.8.3)^109^. EPI-induced geometrical distortions were corrected by warping the diffusion-weighted image volumes to down-sampled T_1_-weighted images with a resolution of 1.5 × 1.5 × 1.5 mm^110^. After preprocessing, the NODDI model^22^ was fitted to the dual-shell HARDI data using fast, linear model fitting algorithms of the AMICO framework^111^. Restricted non-Gaussian diffusion in the intra-axonal space was quantified with ICSF and free isotropic Gaussian diffusion with ISOSF. ODI was modelled with a Watson distribution.

MT-weighted GRE volumes for each participant were co-registered to the MT-volume with the most contrast using a rigid body (6 degrees of freedom) registration to correct for inter-scan motion using Elastix^112^. The 11 MT-weighted GRE images and T1-maps were modelled by the two-pool Ramani’s pulsed MT approximation^113^ yielding MPF, *k*_*f*_, and R_1_ maps. MPF maps were threshholded to an upper intensity limit of 0.3 and *k*_*f*_ maps to an upper limit of 3 using the FMRIB’s fslmaths imaging calculator to remove voxels with noise-only data.

A monoexponential decay function was fitted to the T_2_ images using quimultiecho from the Quantitative Imaging Tools (QUIT) library (http://spinicist.github.io/QUIT). This fitting excluded the first acquired TE due to a non-contained stimulated echo artifact in the first echo of the multi-contrast spin echo Siemens library sequence. R_2_ was calculated as 1/T_2_ in second units.

All image modality maps and region of interest masks were spatially aligned to the T_1_-weighted anatomical volume as reference image with linear affine registration (12 degrees of freedom) using FMRIB’s Linear Image Registration Tool (FLIRT).

Five R_2_ and four qMT data sets were missing as five participants did not complete the MRI session due to claustrophobia.

### Tractography

The RESDORE algorithm^114^ was applied to identify outliers, followed by whole brain tractography with the damped Richardson-Lucy algorithm (dRL)^115^ on the 60 direction, b = 2400 s/mm^2^ HARDI data in single-subject space using in house software^114^ coded in MATLAB (the MathWorks, Natick, MA). To reconstruct fibre tracts, dRL fibre orientation density functions (fODFs) were estimated at the center of each image voxel. Seed points were positioned at the vertices of a 2 × 2 × 2 mm grid superimposed over the image. The dRL algorithm interpolated local fODF estimates at each seed point and then propagated 0.5 mm along orientations of each fODF lobe above a threshold peak of 0.05. This process was repeated until the minimally subtending peak magnitude fell below 0.05 or the change of direction between successive 0.5 mm steps exceeded an angle of 45°. Tracking was then repeated in the opposite direction from the initial seed point.

The fornix and the PHC were reconstructed with an in-house automated segmentation method based on principal component analysis (PCA) of streamline shape^116^. This involved the manual reconstruction of a set of tracts from 20 randomly selected datasets, that were then used to train a PCA model of candidate streamline shape and location. The fornix and the PHC tracts were reconstructed by manually applying region of interest (ROI) gates to isolate specific tracts from the whole brain tractography data on colour-coded fiber orientation maps in ExploreDTI following previously published protocols^21,57,117,118^. The trained PCA shape models were then applied to all datasets: candidate streamlines, i.e. those bridging the gap between estimated end points of the fornix/PHC, were selected from the whole volume tractography and spurious streamlines were excluded by means of a shape comparison with the trained PCA model. All automatic tract reconstructions underwent visual quality control and any remaining spurious fibers that were not consistent with the tract anatomy were removed.

### Whole Hippocampal segmentation

Volumetric segmentation of the left and right whole hippocampi from T_1_- weighted images was performed with the Freesurfer image analysis suite (version 5.3), which is documented online (https://surfer.nmr.mgh.harvard.edu/). Mean intracranial volume fractions (ICV) were extracted for each brain as estimates of individual differences in head sizes. Two datasets had to be excluded from the analysis due to motion artefacts.

### Statistical analyses

Statistical analyses were conducted in MATLAB, SPSS version 20^119^ and the PROCESS computational tool for mediation analysis^120^. Multiple comparisons were corrected with a False Discovery Rate (FDR) of 5% using the Benjamini-Hochberg procedure^121^. Partial Eta^2^ (ηp^2^) and correlation coefficients are reported as indices of effect sizes. Differences in the size of correlation coefficients were tested with the Fisher’s r-to-z transformation^122^. All reported p-values are two-tailed.

Omnibus multivariate regression analysis was conducted to test for main effects of age, genetic and lifestyle risk factors, sex, education and head size simultaneously on all MRI metrics. Omnibus effects were followed up with *post-hoc* multivariate covariance analysis testing for effects on individual MRI metrics in all regions of interest while correcting for any confounding variables. The direction of age effects and the relationship between hippocampal and white matter microstructural metrics were investigated with Pearson correlation coefficients.

Linear mediation analysis was then used to test for the indirect effects a*b of hippocampal mediator variables on the direct effects c’ of age on the fornix metrics (Fig. 1, Model A) and for indirect effects a*b of fornix mediator variables on direct age effects c’ on hippocampal differences (Fig. 1, Model B). The significance of indirect and direct effects was assessed with a 95% confidence interval based on bootstrapping with 5000 replacements^120^.

The impact of genetic and lifestyle risk factors on mediator variables was explored with hierarchical linear regression analyses, and Pearson correlation coefficients between MRI mediator variables and episodic memory measures were calculated to explore brain-cognition relationships.

## Data Availability

The datasets generated during and/or analysed during the current study are available from the corresponding author on reasonable request.
