# Author Correction: Fornix white matter glia damage causes hippocampal gray matter damage during age-dependent limbic decline

**DOI:** 10.1038/s41598-019-51737-1

**Published:** 2019-10-17

**Authors:** Claudia Metzler-Baddeley, Jilu P. Mole, Rebecca Sims, Fabrizio Fasano, John Evans, Derek K. Jones, John P. Aggleton, Roland J. Baddeley

**Affiliations:** 1 Cardiff University Brain Research Imaging Centre (CUBRIC), Maindy Road, Cathays, Cardiff, CF24 4HQ UK; 2 Psychological Medicine and clinical neurosciences, School of Medicine, Cardiff University, Maindy Road, Cathays, Cardiff, CF24 4HQ UK; 3 Siemens Healthcare, Head Office, Sir William Siemens Square, Surrey, GU16 8QD UK; 4 School of Psychology, Faculty of Health Sciences, Australian Catholic University, Melbourne, Victoria, 3065 Australia; 5 School of Psychology, Cardiff University, Tower Building, 70 Park Place, Cardiff, CF10 3AT UK; 6 Experimental Psychology, University of Bristol, 12a Priory Road, Bristol, BS8 1TU UK

Correction to: *Scientific Reports* 10.1038/s41598-018-37658-5, published online 31 January 2019

This Article contains errors in Tables 2 and 3, where the uncorrected p-values are given rather than the corrected Benjamini-Hochberg adjusted p-values. As a result the Table legends,

“Summary of the effects of age on gray and white matter microstructural indices. *Controlled for intracranial volume, **5% FDR corrected. ISOSF = isotropic signal fraction, MPF = macromolecular proton fraction, ODI = orientation dispersion index, PHC = parahippocampal cingulum.”

should read:

“Summary of the effects of age on gray and white matter microstructural indices. *Controlled for intracranial volume, **5% False Discovery Rate Benjamini-Hochberg adjusted p-values. ISOSF = isotropic signal fraction, MPF = macromolecular proton fraction, ODI = orientation dispersion index, PHC = parahippocampal cingulum.”

and

“Summary of the results of the hierarchical regression models testing for the effects of genetic and lifestyle risk variables on fornix and hippocampus mediator variables. 5% FDR corrected p-values are highlighted in bolds.”

should read:

“Summary of the results of the hierarchical regression models testing for the effects of genetic and lifestyle risk variables on fornix and hippocampus mediator variables. p_BHadj_, 5% False Discovery Rate Benjamini-Hochberg adjusted p-values (significant p-values are highlighted in bold). BP = blood pressure, FH = family history, ICV = intracranial volume, ISOSF = isotropic signal fraction, MPF = macromolecular proton fraction, WHR = waist-hip-ratio.”

The correct Tables 2 and 3 appear below with their accompanying legends as Tables 1 and 2 respectively.

**Table 1:** Summary of the effects of age on gray and white matter microstructural indices. *Controlled for intracranial volume, **5% False Discovery Rate Benjamini-Hochberg adjusted p-values. ISOSF = isotropic signal fraction, MPF = macromolecular proton fraction, ODI = orientation dispersion index, PHC = parahippocampal cingulum.

MRI index			F_(2,152)_-value^*^	Benjamini-Hochberg corrected p-value**	Effect size ηp^2^
Fornix		MPF	11.9	0.0002	0.14
		*k*_*f*_	10.0	0.0008	0.12
		R_1_	12.4	0.00035	0.14
		ISOSF	8.9	0.001	0.11
		ODI	5.0	0.03	0.06
Left PHC		R_2_	7.5	0.005	0.09
Right PHC		R_1_	4.7	0.03	0.06
Left hippocampus		*k*_*f*_	6.7	0.008	0.08
		R_1_	5.0	0.025	0.06
		ISOSF	12.2	0.0002	0.14
		ODI	9.8	0.0007	0.12
Right hippocampus		ISOSF	7.5	0.004	0.09

**Table 2:** Summary of the results of the hierarchical regression models testing for the effects of genetic and lifestyle risk variables on fornix and hippocampus mediator variables. p_BHadj_, 5% False Discovery Rate Benjamini-Hochberg adjusted p-values (significant p-values are highlighted in bold). BP = blood pressure, FH = family history, ICV = intracranial volume, ISOSF = isotropic signal fraction, MPF = macromolecular proton fraction, WHR = waist-hip-ratio.

Outcome variables	Adjusted R^2^	Predictors in final regression model
Fornix MPF	0.24 (p < 0.001)	**Age (p**_**BHadj**_ **< 0.001)** **WHR (p**_**BHadj**_ **= 0.045)**
Fornix R_1_	0.28 (p < 0.001)	**Age (p**_**BHadj**_ **< 0.001)** **ICV (p**_**BHadj**_ **= 0.026)** **Alcohol (p**_**BHadj**_ **= 0.03)** **WHR (p**_**BHadj**_ **= 0.04)**
Fornix *k*_*f*_	0.23 (p < 0.001)	**Age (p**_**BHadj**_ **< 0.001)** WHR (p_BHadj_ = 0.07)
Fornix ISOSF	0.32 (p < 0.001)	**Age (p**_**BHadj**_ **< 0.001)** ICV (p_BHadj_ = 0.05) **Sex (p**_**BHadj**_ **= 0.004)**
Right hippocampal ISOSF	0.36 (p < 0.001)	**Age (p**_**BHadj**_ **< 0.001)** **ICV (p**_**BHadj**_ **= 0.026)** **Sex (p**_**BHadj**_ **= 0.007)** Diastolic BP (p_BHadj_ = 0.07) FH (p_BHadj_ = 0.07)

In addition, in Figure 3 the p-values for the following scatterplots are incorrect: Fornix ODI, LPHC R_2_, RPHC R_1_, LHC R_1_, LHC ODI and LHC *k*_*f*_. As a result, the Figure legend,

“Plots the correlations and Pearson coefficients (controlled for intracranial volume) between age and white and gray matter microstructural indices. Abbr.: ICSF = intracellular signal fraction, ISOSF = isotropic signal fraction, *k*_*f*_ = forward exchange rate, LHC = left hippocampus, LPHC = left parahippocampal cingulum, MPF = Macromolecular proton fraction, ODI = orientation dispersion index, R = longitudinal relaxation rate, RHC = right hippocampus, RPHC = right parahippocampal cingulum ****p < 0.0001, ***p < 0.001, **p < 0.01, *p < 0.05, 5% False Discovery Rate”

should read:

“Scatterplots of the correlations and Pearson coefficients (controlled for intracranial volume) between age and white and gray matter microstructural indices. Abbr.: ICSF = intracellular signal fraction, ISOSF = isotropic signal fraction, *k*_*f*_ = forward exchange rate, LHC = left hippocampus, LPHC = left parahippocampal cingulum, MPF = Macromolecular proton fraction, ODI = orientation dispersion index, R = longitudinal relaxation rate, RHC = right hippocampus, RPHC = right parahippocampal cingulum ****p < 0.0001, ***p < 0.001, **p < 0.01 (5% False Discovery Rate Benjamini-Hochberg adjusted p-values).”

The correct Figure 3 and its accompanying legend appears below as Figure 1.

**Figure 1:**
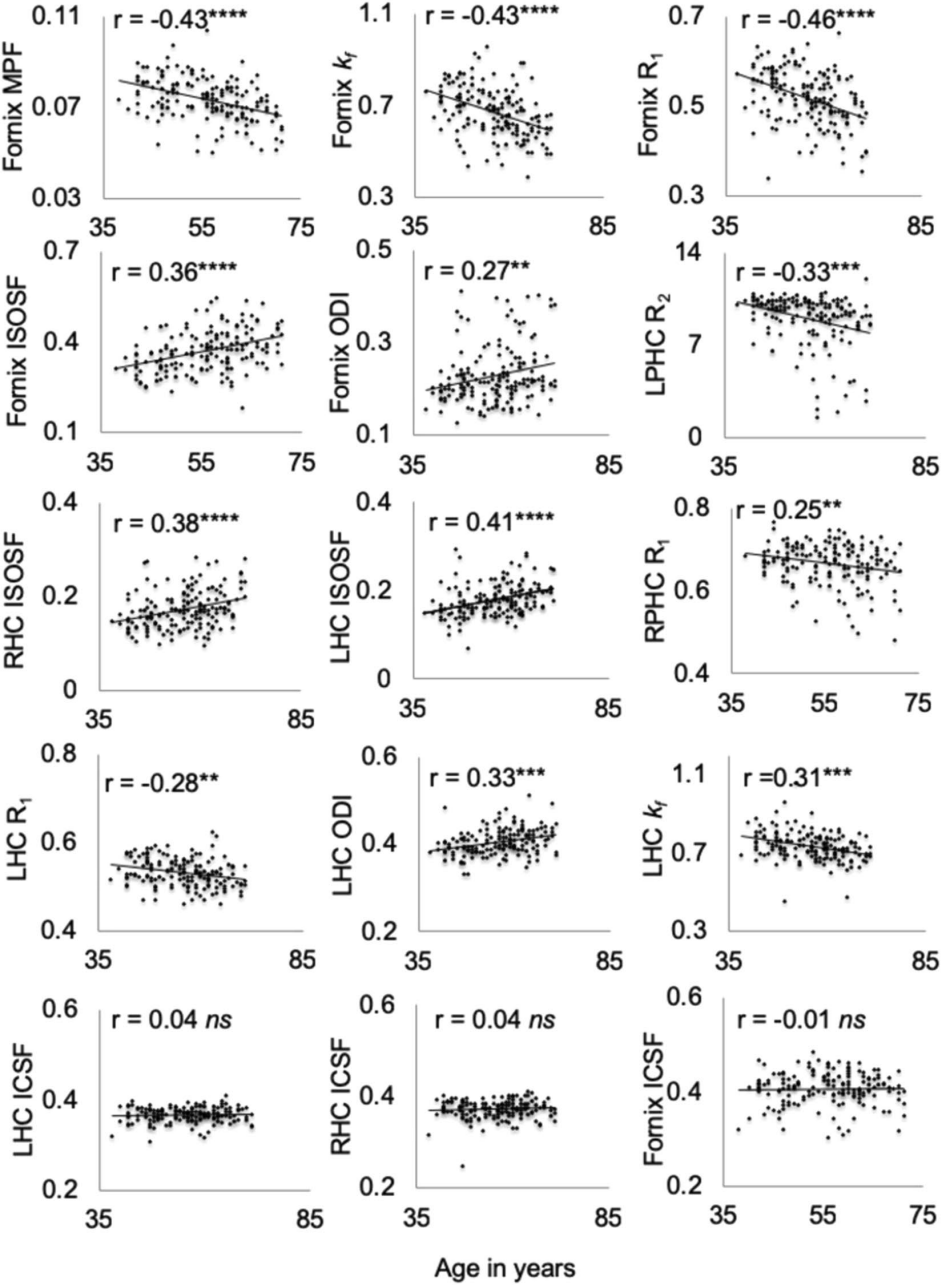
Scatterplots of the correlations and Pearson coefficients (controlled for intracranial volume) between age and white and gray matter microstructural indices. Abbr.: ICSF = intracellular signal fraction, ISOSF = isotropic signal fraction, *k*_*f*_ = forward exchange rate, LHC = left hippocampus, LPHC = left parahippocampal cingulum, MPF = Macromolecular proton fraction, ODI = orientation dispersion index, R = longitudinal relaxation rate, RHC = right hippocampus, RPHC = right parahippocampal cingulum ****p < 0.0001, ***p < 0.001, **p < 0.01 (5% False Discovery Rate Benjamini-Hochberg adjusted p-values).

